# Tailorable Antibacterial Activity and Biofilm Eradication Properties of Biocompatible α-Hydroxy Acid-Based Deep Eutectic Solvents

**DOI:** 10.3390/pharmaceutics18010016

**Published:** 2025-12-22

**Authors:** Gleb Dubinenko, Elena Senkina, Ksenia Golovina, Alexandra Myshova, Olga Igumnova, Evgenii Plotnikov, Arsalan Badaraev, Sven Rutkowski, Victor Filimonov, Sergei Tverdokhlebov

**Affiliations:** 1 Laboratory of Plasma Hybrid Systems, Weinberg Research Center, Tomsk Polytechnic University, 634000 Tomsk, Russia; dubinenko@tpu.ru (G.D.); elena.senkina.1995@mail.ru (E.S.); go_ksenia@mail.ru (K.G.); aem26@tpu.ru (A.M.); olga.igumnova.01@mail.ru (O.I.); adb6@tpu.ru (A.B.); 2 Research School of Chemistry & Applied Biomedical Sciences, Tomsk Polytechnic University, 634000 Tomsk, Russia; plotnikovev@tpu.ru; 3 Mental Health Research Institute, Tomsk National Research Medical Center of the Russian Academy of Sciences, Aleutskaya Street 4, 634014 Tomsk, Russia; 4 Department of Chemistry, Siberian State Medical University, Moskovsky Trakt 2, 634050 Tomsk, Russia; 5 Kizhner Research Center, Tomsk Polytechnic University, 634000 Tomsk, Russia; filimonov@tpu.ru

**Keywords:** deep eutectic solvents, α-hydroxy acids, quaternary ammonium compounds, choline chloride, tetraethylammonium chloride, antibacterial, antibiofilm

## Abstract

**Background/Objectives**: Deep eutectic solvents (DESs) have recently gained attention for their antimicrobial properties, particularly because they target both planktonic bacteria and biofilms. Among these, DESs based on α-hydroxy acids (αHAs) are of interest due to their inherent antibacterial properties and favorable biocompatibility. However, effects of the αHA molecular structure and hydrogen bonding ability within a DES formulation on biological activity has not yet been thoroughly investigated. **Methods**: This study systematically investigates DESs formed by combining glycolic acid, lactic acid or tartaric acid with either choline chloride or tetraethylammonium chloride. **Results**: All DESs demonstrate broad-spectrum antibacterial activity against *Staphylococcus aureus*, methicillin-resistant *Staphylococcus aureus*, *Escherichia coli*, and *Pseudomonas aeruginosa* and effectively inhibit biofilm formation while exhibiting low cytotoxicity toward 3T3-L1 fibroblasts. **Conclusions**: DES formation enhances antibacterial efficacy while attenuating cytotoxicity compared to the individual components, thereby decoupling bactericidal activity from toxicity. Physicochemical characterization confirms the formation of a eutectic phase and reveals that biological activity is primarily governed by acidity rather than by the specific αHA structure or eutectic strength. These results provide new insights into structure-function relationships in DESs and establish a design strategy for biocompatible, non-cytotoxic antimicrobial agents.

## 1. Introduction

Eradication of bacterial biofilms remains a serious clinical challenge, as the extracellular polymeric substance (EPS) matrix ensures mechanical stability of the biofilm and protects the bacteria embedded within it from antimicrobial agents [1,2]. Biofilm-associated infections in wounds, implants, and medical devices are difficult to treat and often persist despite conventional antibiotic therapy [3,4]. There is a clear and urgent need for novel antimicrobial agents that prevent the formation of biofilms, disrupt mature biofilms, eliminate bacteria embedded in these biofilms, and are safe for mammalian cells.

Deep eutectic solvents (DESs) have emerged over the past decade as a promising class of green and environmentally friendly solvents that offer a sustainable alternative to conventional organic solvents and ionic liquids [5,6]. The DESs are defined as mixtures of hydrogen bond donor (HBD) and acceptor (HBA) that form a eutectic phase with significantly lower melting points than their individual components. DESs provide a versatile approach for developing liquid-phase materials from environmentally friendly and often biodegradable substances [6,7]. Due to their adjustable physicochemical properties, ease of preparation, and biocompatibility, DESs have been successfully applied in organic synthesis, extraction, catalysis, separation technologies, electrochemistry, and biomass processing [8,9,10,11].

More recently, DESs have attracted the interest in biomedical research, particularly as potential bioactive compounds with antimicrobial properties [12,13]. Their intrinsic high viscosity, strong hydrogen bonding networks, and the ability to disrupt non-covalent bonds make DESs promising candidates for interacting with and destabilizing bacterial membranes [14]. A variety of DESs consisting of components of different chemical compositions have been investigated with regard to their antibacterial properties [13]. DESs formed from quaternary ammonium compounds (QACs) in combination with various HBDs including organic acids, phenolic acids, alcohols, and sugars have demonstrated the ability to inhibit bacterial growth [13,14,15,16,17,18]. These findings suggest that the antimicrobial activity of DESs can be tailored through selecting specific components. Recent studies have demonstrated the antibacterial potential of DESs prepared from organic acids and QACs [13,18,19,20]. Organic α-hydroxy acids (αHAs) such as glycolic acid (GA), lactic acid (LA), and tartaric acid (TA) are biocompatible and biodegradable compounds with known antimicrobial properties that are widely used in biomedical and cosmetic applications and are capable of forming eutectic mixtures as HBD [21,22,23]. When combined with QACs, which are generally recognized as safe and widely used in pharmaceutical formulations [24,25], αHAs can form eutectic mixtures that retain or even enhance their biological activity [18]. Eutectic formation may stabilize acid groups, alter pH, modify diffusivity, and possibly reduce the cytotoxicity of αHAs [26].

While numerous studies have reported on DESs with antimicrobial properties, limited understanding of how HBDs and HBAs influence biological activity limits their rational development. In particular, the relationship between the molecular structure of HBDs and HBAs, their hydrogen bonding capacity, their acidity, and the resulting antibacterial or antibiofilm efficiency remains barely understood. Moreover, most studies to date have focused either on planktonic bacteria or on physicochemical characterization of DESs alone, without integrating structural, microbiological, and cytocompatibility-related data within a systematic investigation. Therefore, structure-activity relationships in antibacterial DESs, should be determined by combining physicochemical characterization with microbiological evaluation and cell studies. This applies especially for those DESs consisting of clinically relevant αHAs and QACs.

In this study, a series of DESs based on GA, LA, and TA as HBDs and combined with either choline chloride (ChCl) or tetraethylammonium chloride (TEAC) as HBAs were prepared and studied. The selected DESs were comprehensively characterized in terms of their physicochemical properties and hydrogen bonding behavior. In addition, their antibacterial activities against planktonic and biofilm-associated bacterial strains, as well as their cytotoxicity toward fibroblast cells, were investigated. The aim was to identify the key structure-property relationships that regulate the biological performance of αHA-based DESs and to determine whether the formation of eutectics can enhance antibacterial efficacy while reducing cytotoxic effects. The findings aim to provide a rational framework for the development of DES-based antimicrobial agents with tunable bioactivity and biocompatibility.

## 2. Materials and Methods

Choline chloride (ChCl, ≥99% purity), tetraethylammonium chloride (TEAC, ≥98% purity), glycolic acid (GA, 70 wt.% aqueous solution) and L-(+)-tartaric acid (TA; ≥99.5% purity) were obtained from Shanghai Macklin Biochemical Co., Ltd. (Shanghai, China). L-(+)-lactic acid (LA, 85 wt.% aqueous solution) was purchased from Acmec Biochemical (Shanghai, China).

### 2.1. Preparation of Deep Eutectic Solvents (DESs)

Prior to use, ChCl and TEAC were dried under vacuum (1 mbar) at room temperature for 24 h using a VTSH-K24-250 (Aktan, Moscow, Russia) vacuum oven. Glycolic acid (70 wt.% aqueous solution) and lactic acid (85 wt.% aqueous solution) were used as supplied. For tartaric acid, an 80 wt.% aqueous solution was prepared by dissolving the powder in deionized water (ISO 3696; Niagara Manufacturing and Commercial Company, Nizhny Novgorod, Russia) [27].

Deep eutectic solvents (DESs) were prepared by mixing the selected α-hydroxy acid (HBD) with the quaternary ammonium salt (HBA) in a molar ratio of 2:1 (HBD:HBA) using a round-bottom flask. The mixtures were stirred at 80 °C for 6 h until homogeneous liquids were obtained. All DESs formulations were stored in sealed containers at room temperature and used without further purification.

### 2.2. Methods of Investigations

#### 2.2.1. Differential Scanning Calorimetry

Differential scanning calorimetry (DSC) was performed using a DSC600 system (Linkam Scientific Instruments, Salfords, UK) to investigate the thermal behavior of the DESs. Approximately 10 mg of each sample was placed in a standard aluminum crucible and hermetically sealed. All measurements were carried out in an argon atmosphere. The samples were first heated from room temperature to 80 °C at a rate of 5 °C·min^−1^ and then cooled to −40 °C at a rate of 1 °C·min^−1^.

#### 2.2.2. Density and Dynamic Viscosity

The density and dynamic viscosity of the DESs prepared were measured using an automatic kinematic viscometer (Stabinger Viscometer™ Series SVM 3000, Anton Paar, Graz, Austria) equipped with a temperature-controlled measurement cell. All measurements were performed in triplicate in the temperature range from 0 °C to 80 °C in increments of 5 °C. At each temperature point, the samples were equilibrated for at least 5 min prior to measurement to ensure thermal stability.

#### 2.2.3. Surface Tension

The surface tension of the DESs formulations was measured using the Du Noüy ring method with a tensiometer (Krüss EasyDyne K20, Krüss GmbH, Hamburg, Germany). A platinum-iridium ring with a diameter of 9.545 mm was used for all measurements. Each sample was equilibrated at 37 ± 1 °C prior to measurement. Surface tension values were determined from the average of three independent measurements for each group.

#### 2.2.4. Fourier Transform Infrared Spectroscopy

The chemical structure and hydrogen bonds within the DES formulations were analyzed using Fourier transform infrared (FTIR) spectroscopy. All spectra were recorded with a Tensor 27 spectrometer (Bruker Optik GmbH, Ettlingen, Germany) equipped with a Miracle™ attenuated total reflection (ATR) attachment (PIKE Technologies, Madison, WI, USA). A ZnSe crystal with an incidence angle of 45° was used as the ATR element. A resolution of 4 cm^−1^ was used to record the spectra in the range from 650 cm^−1^ to 4000 cm^−1^.

#### 2.2.5. pH Measurement

The corresponding acidity of the DES samples was evaluated by potentiometric pH measurement. For this purpose, 100 mM aqueous solutions of each DES were prepared using deionized water. Measurements were carried out using a TAN-1 pH meter (NPP Tomanalit, Tomsk, Russia) equipped with a STMICRO8 pH electrode (OHAUS Corporation, Changzhou, China). The pH electrode was calibrated prior to measurement using standard buffer solutions with pH values from 1.65 to 6.86 (EcoUnit, Moscow, Russia). All measurements were performed at room temperature ((22 ± 1) °C), and each value was determined as the average of at least three independent readings.

#### 2.2.6. Antibacterial Activity of DESs (Agar Diffusion Test)

Antibacterial activity of the DESs was assessed using the agar diffusion test against both Gram-positive and Gram-negative bacterial strains. The following strains were used: *Staphylococcus aureus* ATCC 6538-P (*S. aureus*), methicillin-resistant *Staphylococcus aureus* ATCC 43300 (MRSA), *Escherichia coli* ATCC 25922 (*E. coli*), and *Pseudomonas aeruginosa* ATCC 27853 (*P. aeruginosa*). GRM-agar nutrient medium (FBUNC PMB, Obolensk, Russia) was poured into sterile 90 mm plastic Petri dishes and left to solidify on a horizontal surface. After solidification, the bacterial suspensions, whose density was adjusted to 0.5 McFarland standard (approx. 5·10^8^ CFU·mL^−1^) were evenly distributed over the surface using a sterile microbiological spreader. Cellulose disks with a diameter of 6 mm were placed on the agar surface, and 20 μL of each DES formulation was carefully added on a disk surface using sterile plastic pipette tips. To ensure consistent diffusion conditions, all plates were pre-incubated at room temperature for (1–2) h prior to incubation at 37 °C for 24 h under aerobic conditions. After incubation, the diameters of inhibition zones were measured with a precision of ±1 mm. Ampicillin (25 mg; Oxoid™, Thermo Scientific, UK) and ceftazidime (25 mg; Krasfarma, Russia) antimicrobial susceptibility discs were used as positive controls for Staphylococcus aureus (including MRSA) and Escherichia coli, and for Pseudomonas aeruginosa, respectively. Positive controls were included for each bacterial strain. Ampicillin discs Oxoid™ (25 mg; Thermo Scientific, Altrincham, UK) for *S. aureus*, MRSA and *E. coli*, and ceftazidime discs (25 mg; Krasfarma, Krasnoyarsk, Russia) for *P. aeruginosa*.

#### 2.2.7. Minimum Inhibitory Concentration (MIC) and Minimum Bactericidal Concentration (MBC) of DESs

The minimum inhibitory concentration (MIC) of the DES formulations was determined according to the established protocol with minor modifications [28] by the broth microdilution method, whereby the optical density was measured at 630 nm instead of 600 nm due to the instrumental configuration of the microplate reader used. The bacterial strains used in this assay were *Staphylococcus aureus* ATCC 6538-P (*S. aureus*), methicillin-resistant *Staphylococcus aureus* ATCC 43300 (MRSA), *Escherichia coli* ATCC 25922 (*E. coli*), and *Pseudomonas aeruginosa* ATCC 27853 (*P. aeruginosa*). Serial dilutions of each DES were prepared in sterile 96-well flat-bottom microplates using Mueller-Hinton broth (MHB; FBIS “SRCAMB”, Obolensk, Russia). The initial DES concentration was 100 mg·mL^−1^. Bacterial inoculum was prepared from 24-h cultures grown on Mueller-Hinton agar (MHA; FBIS “SRCAMB”, Obolensk, Russia). The colonies were suspended in sterile MHB to a turbidity equivalent to the 0.5 McFarland standard (approximately 1.5·10^8^ CFU·mL^−1^) and then diluted in MHB to yield a final concentration of ~1.5·10^5^ CFU·mL^−1^. A volume of 100 μL of this suspension was added to each well containing the DES dilution. The well plates were sealed and incubated at 37 °C for 24 h. Bacterial growth was evaluated by measuring the optical density at 630 nm using an iMark™ microplate absorbance reader (Bio-Rad Laboratories, Inc., Hercules, CA, USA). Negative controls (bacteria without DES) and sterility controls (medium without bacteria) were included on each plate. MIC was defined as the lowest DES concentration at which no visible growth was observed and the optical density (OD) remained at the baseline value. MIC values for antibiotics were taken from the EUCAST database and are for reference only.

To determine the minimum bactericidal concentration (MBC), aliquots from wells showing no visible growth were plated on MHA. After 24 h of incubation at 37 °C, the lowest DES concentration at which no bacterial colonies appeared was recorded as the MBC. As expected, the MBC values were typically 1 to 3 dilution steps above the corresponding MIC values.

#### 2.2.8. Time-Kill Kinetics Assay

Time-kill kinetics were evaluated for each DES formulation at a concentration corresponding to its minimum inhibitory concentration (1 × MIC). DES samples were diluted in Mueller–Hinton broth (MHB; FBIS “SRCAMB”, Obolensk, Russia) to a final volume of 10 mL in sterile 15 mL polypropylene tubes. An overnight bacterial culture was adjusted to an OD_600_ corresponding of 1.5 × 10^8^ CFU·mL^−1^ (0.5 McFarland standard) and subsequently diluted to yield an initial inoculum of approximately 5 × 10^5^–1 × 10^6^ CFU·mL^−1^. A 33 µL aliquot of this suspension was added to each tube, followed by gentle vortexing and incubation at 35 °C. At predetermined time points (0, 1, 2, 4, 6, and 24 h), 200 µL aliquots were withdrawn, serially diluted in sterile 0.9% NaCl solution, and plated on MHA. The plates were incubated at 37 °C for 24 h, after which the surviving colonies were counted and recorded as CFU·mL^−1^. The lower limit of detection (LOD) for the assay was 1.0 log_10_ CFU·mL^−1^, and the bactericidal activity was defined as a 3-log (99.9%) reduction in viable cells relative to the initial inoculum. All experiments were performed in triplicate.

#### 2.2.9. Cytotoxicity Assay

The cytotoxicity of the DES formulations was evaluated using an MTT assay with mouse embryonic fibroblast cells (3T3-L1 line). Cells were seeded at a density of 5000 cells per well into sterile flat-bottom 96-well plates (SPL Life Sciences Co., Ltd., Pocheon, Republic of Korea). Bacterial cells were allowed to adhere and proliferate for 24 h, resulting in a reproducible confluence of approximately 70% confluence at the time of experiment. All experiments were performed on bacterial cell cultures in the growth phase with a comparable confluence of at least 70% to minimize variability. Cell counting was performed using an automated cell counter (Countess™ 2 FL, Thermo Fisher Scientific Inc., Waltham, MA, USA). After 24 h of incubation, the culture medium was replaced with fresh medium containing DESs at an initial concentration of 50 mg·mL^−1^, followed by serial two-fold dilutions. The cells were incubated under standard culture conditions (37 °C, 5% CO_2_, humidified atmosphere) in an incubator (CB-170, Binder GmbH, Tuttlingen, Germany) for an additional 24 h. Cell viability was assessed by replacing the medium with a 0.45 mg·mL^−1^ solution of 3-(4,5-dimethylthiazol-2-yl)-2,5-diphenyl tetrazolium bromide (MTT; PanEco LLC, Moscow, Russia), followed by incubation for 4 h at 37 °C in 5% CO_2_. After incubation, the MTT solution was removed and 100 μL of dimethyl sulfoxide (DMSO; PanEco LLC, Moscow, Russia) was added to each well to dissolve the formazan crystals. The optical density was measured at 570 nm using a Multiskan FC microplate photometer (Thermo Fisher Scientific Inc., Waltham, MA, USA). Cell viability was calculated as a percentage relative to untreated controls. IC_50_ values (half-maximal inhibitory concentrations) were determined from dose-response curves fitted using nonlinear regression analysis.

#### 2.2.10. Biofilm Inhibition Assay

The ability of DESs to inhibit biofilm formation was evaluated using *Pseudomonas aeruginosa* ATCC 27853 (*P. aeruginosa*) as a model organism. A bacterial suspension was prepared in Mueller-Hinton broth (MHB; FBIS “SRCAMB”, Obolensk, Russia) with a turbidity equivalent to the 0.5 McFarland standard (approximately 1.5·10^8^ CFU·mL^−1^) and subsequently diluted 1:100 in fresh MHB. Aliquots of 100 μL of the diluted suspension were added to sterile 96-well flat-bottom microplates (SPL Life Sciences Co., Ltd., Pocheon, Republic of Korea), followed by the addition of 100 μL of DES solutions in MHB at final concentrations ranging from 0.5 to 50 mg·mL^−1^. The microplates were incubated for 24 h at 37 °C under static conditions to allow biofilm formation. After incubation, the wells were gently washed twice with phosphate-buffered saline (PBS; pH 7.4; Rosmedbio, Saint-Petersburg, Russia) to remove non-adherent (planktonic) cells. The biofilm biomass was stained with 0.25% crystal violet (Minimed, Suponevo, Russia) in 95% ethanol (Samaramedprom, Samara, Rusian Federation) by adding 100 μL to each well and incubating at room temperature for 15 min. Excess dye was removed by rinsing with PBS, and the residual crystal violet was solubilized by adding 100 μL of 95% ethanol. Subsequently, the optical density (OD) of each well was measured at 630 nm using a Multiskan™ FC microplate reader (Thermo Fisher Scientific Inc., Waltham, MA, USA). Biofilm inhibition was calculated as the percentage reduction in OD relative to untreated control wells containing bacteria without DES. All OD measurements were performed in triplicate.

#### 2.2.11. Biofilm Eradication Assay

The ability of DES formulations to eradicate established biofilms was assessed using *Pseudomonas aeruginosa* ATCC 27853 (*P. aeruginosa*). Initially, the bacteria were diluted in PBS to a turbidity equivalent of 0.5 according to the McFarland standard (approximately 1.5 × 10^8^ CFU·mL^−1^). Next, a bacterial suspension was prepared in MHB with a final concentration of 1.5 × 10^7^ CFU·mL^−1^. Mature biofilms were prepared by incubating bacterial suspensions in sterile 96-well flat-bottom plates (TPP Techno Plastic Products AG, Trasadingen, Switzerland) at 37 °C for 48 h. After incubation, the wells were washed again with PBS to remove planktonic cells and detached biomass. Subsequently, DES solutions in MHB at concentrations ranging from 10 to 400 mg·mL^−1^ were added to each well. Plates were incubated for 24 h at 37 °C under constant conditions. After incubation, the wells were rinsed with PBS to remove any residual material and exfoliated biomass. The remaining biofilms were stained with 200 µL of a 1% aqueous solution of crystal violet (Minimed, Suponevo, Russia) for 20 min at room temperature. Excess stains were removed by washing three times with PBS, and the bound dye was solubilized with 200 µL of 95% ethanol. The optical density (OD) of each well was measured at 630 nm using a Multiskan™ FC microplate reader (Thermo Fisher Scientific Inc., Waltham, MA, USA). Biofilm eradication efficiency was calculated as a percentage using the following equation:

(1) $$Biofilm\;Eradication\left(\%\right)=\frac{\left({OD}_{control}-{OD}_{sample}\right)}{{OD}_{control}}\cdot 100\%,$$

where OD_control_ is the optical density of the untreated biofilm and OD_sample_ is the optical density after treatment with DES.

#### 2.2.12. Biofilms Morphology

The morphological structure of *Pseudomonas aeruginosa* ATCC 27853 (*P. aeruginosa*) biofilms, both untreated and treated with DESs, was examined using scanning electron microscopy (SEM). Biofilms were grown on sterile polymer substrates (Support Information (SI) Figure S1) made of biocompatible photocurable polymeric resin (Dental Clear PRO, HARZ Labs, Moscow, Russia) using a Photon Mono 2 (Anycubic, Shenzhen, China) Liquid Crystal Display (LCD) 3D-printer. The choice of substrate design was based on the findings of Childs et al. [29]. Sterilized substrates were glued to the lid of 24-well cell culture plates (SPL Life Sciences Co., Ltd., Pocheon, Republic of Korea) with the use of cyanoacrylate adhesive CA-500.200 (Weiss Chemie + Technik GmbH & Co. KG., Haiger, Germany). The substrates were immersed into the wells by placing the lid on the plate and then incubated with bacterial suspensions for 48 h at 37 °C under static conditions to allow biofilm formation. After incubation, the substrates were gently rinsed with PBS to remove non-adherent cells. The biofilms were then treated with DES solutions at concentrations ranging from 0.5 mg·mL^−1^ to 400 mg·mL^−1^ under the same incubation conditions for 24 h. After treatment, the samples were rinsed again with PBS and fixed for 30 min at room temperature in 2.5% glutaraldehyde (Sigma-Aldrich, St. Louis, MO, USA) prepared in distilled water (Aqualab AL-1, Aqualab, Moscow, Russia). The fixed samples were washed three times with PBS and dehydrated in a graded ethanol series, followed by air-drying under sterile conditions. Prior to imaging, the substrates were detached from the lid and sputter-coated with a thin conductive layer of gold using a JEOL Smart Coater (JEOL Ltd., Tokyo, Japan) vacuum sputter coater. SEM imaging was performed using a Quanta 200 3D scanning electron microscope (FEI Company, Hillsboro, OR, USA) operated in high vacuum mode at an accelerating voltage of 15 kV. The SEM micrographs were taken at magnifications of 5000× to 40,000× in order to assess the biofilm architecture and morphology of the bacterial cells.

### 2.3. Statistical Analysis

All experiments were performed in independent replicates, as indicated in the corresponding sections. Quantitative data are presented as mean ± standard deviation (SD). Statistical analysis was carried out using OriginPro 2023 (v10.0.0.154, OriginLab Corporation, Northampton, MA, USA). Comparisons between multiple groups were performed using one-way analysis of variance (ANOVA) with Tukey correction and a confidence interval of 95%. Differences were considered statistically significant at *p* < 0.05. Image processing and analysis were conducted using ImageJ (v1.54g, National Institutes of Health, Bethesda, MD, USA).

## 3. Results and Discussion

### 3.1. Physicochemical Characterization

**Figure 1:**
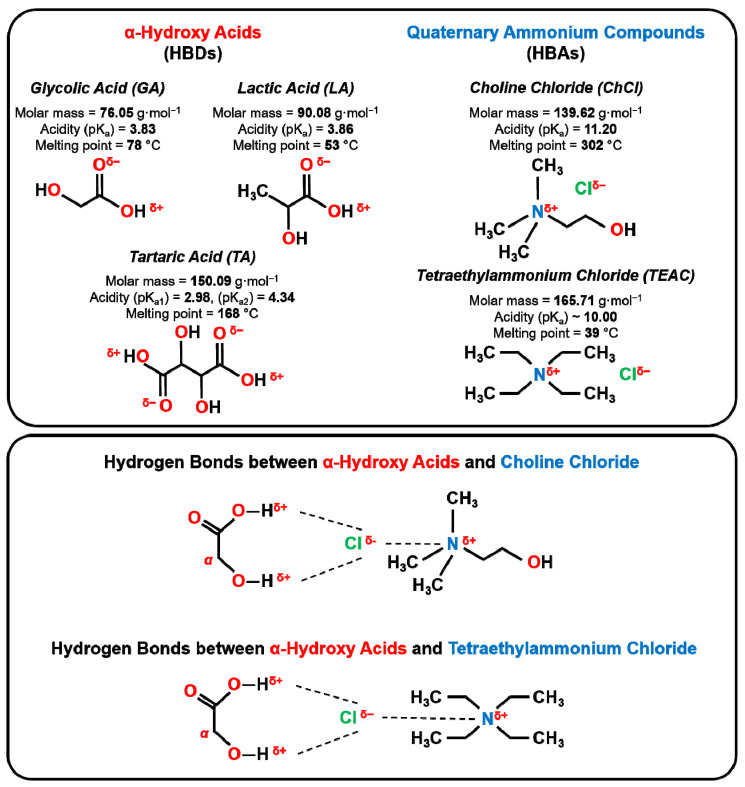
Top row: Chemical structures of hydrogen bond donors (HBDs—α-hydroxy acids: glycolic acid, L-(+)-lactic acid and L-(+)-tartaric acid) and hydrogen bond acceptors (HBAs—quaternary ammonium compounds: choline chloride and tetraethylammonium chloride) used in the preparation of DESs in this study. Bottom row: Common hydrogen bonds between α-hydroxy acids and choline chloride, as well as α-hydroxy acids and tetraethylammonium chloride.

Glycolic acid (GA), L-(+)-lactic acid (LA) and L-(+)-tartaric acid (TA) are α-hydroxy acids (αHAs) characterized by a hydroxyl group in the α-position relative to the carboxylic group (Figure 1). Despite this similar structural feature, these molecules differ in terms of size, the number of functional groups they contain, and their conformational flexibility. These are factors that have a decisive influence on the formation of hydrogen bonds (HBs) and thus on the physicochemical characteristics of deep eutectic solvents (DESs) [30]. It should be noted that HBs differ in their bond length (>2.2 Å for a weak HB to ≤1.2 Å for a strong HB) [31] and their bond strength (very weak HB—(1–2) kJ·mol^−1^ to very strong HB > 155 kJ·mol^−1^) [32]. Moreover, HBs are categorized in intermolecular HBs [33], which take place between molecules and can be homo intermolecular HBs (occurs between one type of molecule) and hetero intermolecular HBs (occurs between different molecule types), as well as intramolecular HBs that occur within a molecule itself [34].

Choline chloride (ChCl) and tetraethylammonium chloride (TEAC) were selected as hydrogen bond acceptors (HBAs; Figure 1). ChCl is widely used in DES design due to its dual hydrogen bonding capability across both the chloride anion and the hydroxyl group [35]. These features enable strong, multivalent interactions with αHAs, thereby stabilizing the DES structure and enhancing its physicochemical properties [36]. In contrast, TEAC lacks additional functional groups beyond the chloride ion, thus it is expected to form weaker hydrogen bond frameworks. This may result in lower viscosity and reduced thermal and phase stability in the corresponding DESs. Moreover, TEAC has been reported to exhibit systemic toxicity in vivo [37]. Its cationic structure is more lipophilic and membrane-active compared to choline, suggesting a higher cytotoxic potential [38]. These features make TEAC a structurally and functionally distinct comparator for evaluating the impact of the HBA on DES properties. Overall, the systematic variation of both the HBD compounds (GA, LA, TA) and the HBA (ChCl, TEAC) compounds provides a rational basis to evaluate how the molecular structure and hydrogen bonding capacity affects the physicochemical and biological properties of the resulting DESs.

Six DESs were prepared by combining one of three αHAs (GA, LA, TA) with either ChCl or TEAC (Table 1). After preparation, all DESs were stored at room temperature ((21 ± 2) °C) for 24 h to assess their phase behavior under ambient conditions. Visual inspection reveals that five formulations are homogeneous clear liquids (SI Figure S2), while TA-TEAC exhibits crystallization, indicating insufficient interaction between the components to maintain a stable eutectic phase. Despite the high number of hydroxyl and carboxyl groups in TA, its interaction with TEAC appears to be too weak to prevent phase separation [39]. This may be attributed to the limited hydrogen bonding capability of TEAC, and therefore weak hydrogen bonds between TEAC and TA.

**Figure 2:**
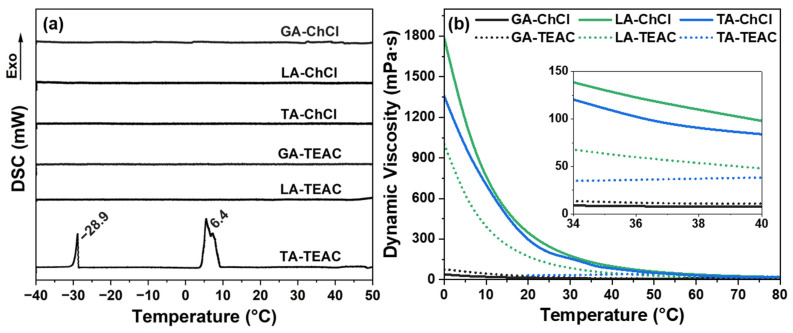
Thermal and rheological properties of the DES formulations under study: (**a**) DSC thermograms of cooling of the DESs and (**b**) temperature-dependent dynamic viscosity profiles of the DESs. The inset in (**b**) displays the temperature range between 34 °C to 40 °C, which indicates the physiologically relevant temperature zone.

To further assess the phase stability, a thermal analysis was performed using differential scanning calorimetry (DSC) (Figure 2a). The cooling thermograms reveal distinct thermal behavior among the DESs tested. Remarkably, only the TA-TEAC formulation exhibits pronounced exothermic transitions upon cooling. Such transitions are observed at approximately (5–10) °C and (−28–−30) °C. These exothermic peaks indicate crystallization processes, suggesting incomplete lowering of the freezing point and the presence of phase-separated or partially crystalline domains [40]. In contrast, under identical DSC conditions, no exothermic or endothermic transitions are observed in the other DES formulations. This indicates a lack of crystallization and implies the formation of kinetically stable, amorphous eutectic mixtures [41]. The observed behavior of TA-TEAC indicates insufficient stabilization of the hydrogen bond framework between TA and TEAC, possibly due to molecular geometry or weaker hydrogen bonding affinity compared to other HBD-HBA combinations [42]. Moreover, these findings are consistent with visual observations of phase separation in TA-TEAC at room temperature (SI Figure S2).

Viscosity of the deep eutectic solvents (DESs) prepared was assessed in the temperature range of 0 °C to 80 °C to evaluate the rheological behavior of each DES formulation (Figure 2b). Similar trends have been reported by Lin et al. [43] in studies of the density and viscosity of DESs. For example, by Ghaedi et al. [44] in studies on temperature-dependent viscosity and by Fan et al. [45] in analyses using Arrhenius behavior. However, the extent of viscosity reduction varied significantly depending on the composition of the DES formulation. Among the DES formulations studied, LA-ChCl displays the highest dynamic viscosity across the entire temperature range, exceeding 1800 mPa·s at 0 °C and declining upon heating. TA-ChCl and LA-TEAC also exhibit relatively high dynamic viscosities, although to a lesser extent. These findings indicate the formation of numerous hydrogen bonds (HBs). Especially for formulations where both the hydrogen bond donor (HBD) and hydrogen bond acceptors (HBA) contain multiple functional groups that can form strong HBs. In contrast, GA-ChCl, GA-TEAC, and TA-TEAC exhibited significantly lower viscosities [46]. The consistently low dynamic viscosity values observed for GA-containing DES may be attributed to weaker hydrogen bonding, higher water content, or lower molecular weight of GA, which may reduce the interaction density between HBD (GA) and the HBA (ChCl or TEAC) [47,48,49]. Notably, the viscosity of TA-TEAC could not be measured due to the onset of crystallization below 20 °C. Furthermore, this formulation exhibits an increase in viscosity at temperatures above 50 °C. That might indicate a phase transition associated with TA precipitation and structural disruption of the eutectic mixture. This behavior supports the earlier hypothesis that TA-TEAC does not form a thermodynamically stable DES. Presumably, this is due to steric hindrance from larger molecular fragments and the limited hydrogen bond capacity of TEAC. The comparison in the physiologically relevant temperatures range ((34–40) °C), presented in the inset of Figure 2b, indicates that all DESs have viscosities below 150 mPa·s at 37 °C. Comparable trends are reported for ChCl-LA formulations by Sazali et al. [50]. Where viscosities remain moderate at (35–40) °C but are higher than analogous systems with weaker HBA. Within this temperature range, the influence of the HBA becomes more pronounced: DESs based on ChCl, particularly those containing LA or TA, exhibit considerably higher viscosities than those based on TEAC. This observation underscores the stronger hydrogen bond frameworks formed by ChCl compared to TEAC.

To support the viscosity measurements and further substantiate the formation of DES phases, a surface tension analysis was conducted. Surface tension serves as a sensitive indicator of molecular organization in liquids and is particularly valuable for distinguishing hydrogen-bonded DESs from simple aqueous solutions of their individual constituents [51,52]. As reported in the literature, DESs progressively lose their eutectic character when the water content exceeds a critical threshold, typically around (0.9–1.0) M, resulting in surface tension values close to those of pure water (~72 mN·m^−1^) [53]. Since all DESs examined in this study contained water, surface tension data are essential to evaluate the persistence of hydrogen bond frameworks under experimental conditions [53]. Among the six DES formulations, the GA-based DESs exhibit the highest surface tension values of (56.43 ± 0.11) mN·m^−1^ for GA-ChCl and (59.05 ± 0.08) mN·m^−1^ for GA-TEAC (Table 2). These elevated values are consistent with the higher water content in these formulations, which also correlates with their lower viscosities and weaker hydrogen bonds. Similarly, TA-TEAC displays a high surface tension ((54.22 ± 0.10) mN·m^−1^). This is consistent with previous observations of limited eutectic stabilization and weak intermolecular interactions in this formulation. In contrast, LA-ChCl, LA-TEAC and TA-ChCl exhibited significantly lower surface tension values, ranging from 43 mN·m^−1^ to 45 mN·m^−1^. These values are markedly lower than those of the GA-based formulations and correspond well with their higher dynamic viscosity values (Figure 2b). The observed inverse correlation between surface tension and viscosity supports the interpretation that stronger hydrogen bond frameworks increase bulk viscosity while simultaneously reducing surface activity [54]. Although water tends to reduce surface tension, none of the DES formulations tested approached the surface tension value of pure water under ambient conditions. Indicating that DESs retain distinct intermolecular interactions. These results suggest that the HBs in the DESs are sufficiently strong to differentiate them from simple aqueous solutions.

**Figure 3:**
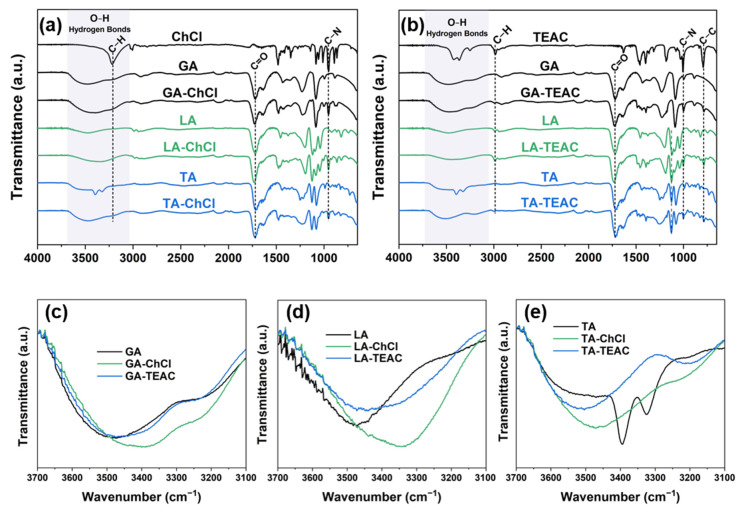
FTIR spectra of hydrogen bonds in DES formulations under investigation: (**a**) FTIR spectra of the individual compounds glycolic acid (GA), L-(+)-lactic acid (LA), L-(+)-tartaric acid (TA), choline chloride (ChCl) and the corresponding DES formulations based on choline chloride, (**b**) FTIR spectra of individual components (GA, LA, TA, TEAC (tetraethylammonium chloride)) and the corresponding DES formulations based on tetraethylammonium chloride, (**c**–**e**) enlarged O-H stretching regions ((3700–3100) cm^−1^) for the DES formulations based on (**c**) GA, (**d**) LA and (**e**) TA.

Fourier-transform infrared (FTIR) spectroscopy was employed to investigate the chemical interactions and evaluate the formation of hydrogen bonds in the DESs prepared (Figure 3). The spectral analysis focuses on evaluating the regions associated with the most important functional groups of the individual compounds, as well as their alterations during DESs formation. ChCl and TEAC exhibit characteristic absorption bands in the region of (3200–2900) cm^−1^, corresponding to C-H stretching vibrations, as well as intense bands between (1100–1000) cm^−1^, associated with C-N stretching modes (Figure 3a,b) [55]. These bands are present in all DES spectra, indicating the retention of molecular integrity of the QACs within the eutectic mixtures. In the case of αHAs, the following two characteristic bands are evident: intense C=O stretching bands in the (1750–1690) cm^−1^ range and broad O-H stretching bands in the range of (3700–3100) cm^−1^ [56]. The latter are particularly sensitive to hydrogen bonding and water content, whereby both band broadening and a shift toward lower wavenumbers indicate enhanced hydrogen bonding [57]. In DES formations with ChCl as HBA, the strongest broadening and shift of the O-H band is observed in the LA-ChCl (Figure 3d). This is indicative of strong intermolecular hydrogen bonds. This is consistent with the high viscosity and low surface tension of this formulation, pointing to a liquid with a dense hydrogen bond framework [58]. In contrast, GA-ChCl and TA-ChCl DESs show moderate band broadening and shifting (Figure 3c,e), suggesting hydrogen bonds of moderate strength. A similar pattern is evident in DESs formed with TEAC (Figure 3b). Among these, LA-TEAC again exhibits the most pronounced O-H band shift and broadening (Figure 3d), indicating enhanced interaction between LA and TEAC. Conversely, GA-TEAC and TA-TEAC display only minor spectral changes relative to the initial acids of these DES formations (Figure 3c,e), indicating smaller hydrogen bond frameworks. This aligns well with other physicochemical data, including the lower viscosity and higher surface tension of these DES formulations.

To assess the acidic environment of the DES formulations, pH measurements were performed. The measured pH values of all DES formulations confirm their strongly acidic nature, with values ranging between 1.98 and 2.31 (Table 2). GA-based DESs exhibit the highest pH values among the studied formulations, with GA-ChCl and GA-TEAC showing pH values of 2.29 ± 0.04 and 2.31 ± 0.02, respectively. In contrast, TA-based formulations demonstrate the lowest pH values, reaching 2.03 ± 0.02 for TA-ChCl and 1.98 ± 0.01 for TA-TEAC. LA-based DESs have an intermediate acidity, with pH values of 2.17 ± 0.02 for LA-ChCl and 2.09 ± 0.01 for LA-TEAC. These variations correspond to the intrinsic acidity of the αHA donors, with TA (pKa_1_ ~3.0, see Figure 1) being more acidic than LA (pKa ~3.9, see Figure 1) and GA (pKa ~3.8, see Figure 1). These observations are in line with earlier studies showing that DESs composed of carboxylic acid-based HBDs exhibit pH values in the range of (1.0–3.0), depending on their composition and water content [59].

### 3.2. Biological Characterization

The antibacterial properties of the DESs formulation prepared were evaluated using the disk diffusion method. Antimicrobial activity was assessed against two Gram-positive strains *S. aureus* and methicillin-resistant *S. aureus* and two Gram-negative strains *E. coli* and *P. aeruginosa*. The resulting inhibition zones are presented in supplementary manuscript (SI Figure S3). DESs of all groups produced inhibition zones comparable in diameter to the zones produced by the conventional antibiotics ampicillin and ceftazidime (Table 3). Despite the fact that the well diffusion assay is qualitative and may be influenced by the physicochemical properties of the test compounds [60], clear trends are observed. In general, the antibacterial activity of the DESs correlates with their viscosity. Although the inhibition zone diameters observed for DES formulations are comparable to those obtained with antibiotics, a direct quantitative comparison is not appropriate. In the disk-diffusion assay, the DESs are applied in their pure form, while the antibiotics are tested at a fixed dose of 25 mg per disk. Therefore, the similarity in the sizes of the inhibition zones reflects differences in formulation, applied mass, and diffusion behavior rather than equivalent intrinsic antibacterial efficacy. A comparison of the sizes of the inhibition zones produced by DESs, LA-TEAC, and TA-ChCl formulations shows that the smallest inhibition zones are formed by the TA-ChCl formulation. This is consistent with their relatively high viscosity, leading to limited diffusion through agar [18,61]. In contrast, formulations based on GA and TA-TEAC, which exhibit lower viscosity values in the measurements, show substantially larger inhibition zones, suggesting that the diffusion rate is an important factor. It is noteworthy that the LA-ChCl formulation displays a pronounced antibacterial activity despite its viscosity being comparable to that of LA-TEAC and TA-ChCl formulations. This observation suggests that other factors such as pH, molecular size, and interaction with bacterial membranes may further modulate the observed activity [62,63]. The literature on carboxylic acids confirms that stronger acidity and multiple acidic functionalities correlate with enhanced antibacterial activity [64]. Importantly, all DES formulations tested display consistent inhibitory activity against both Gram-positive and Gram-negative strains, highlighting their broad-spectrum antibacterial potential.

The antibacterial properties of the DES formulations were evaluated by determining their minimum inhibitory concentration (MIC) and minimum bactericidal concentration (MBC) against both Gram-positive (*S. aureus*, MRSA) and Gram-negative (*E. coli*, *P. aeruginosa*) strains (Table 4). The αHAs exhibit lower MIC values than the corresponding DES formulations, confirming their inherent antibacterial potential due to their low pH and acid-induced membrane stress. However, the DESs show comparable or only slightly higher MIC and MBC values. This indicates that eutectic formation does not suppress antibacterial activity but rather modulates it through changes in molecular organization. Among the ChCl-based formulations, GA-ChCl and LA-ChCl exhibit relatively high MIC values (9.06–9.08) mg·mL^−1^ and MBC values of up to 36.25 mg·mL^−1^, indicating moderate antibacterial activity without pronounced selectivity between Gram-positive and Gram-negative strains (Table 4). TA-ChCl shows significantly improved activity with MIC and MBC values of 4.97 mg·mL^−1^ and 9.92 mg·mL^−1^, respectively. This enhanced activity may be attributed to the higher acidity and the presence of two carboxyl groups in TA, potentially facilitating membrane disruption or pH-dependent stress mechanisms [13]. Substituting ChCl with TEAC as the HBA further increased antibacterial efficacy in all αHA-based DESs formulations (Table 4). All TEAC-based formulations exhibit lower MIC and MBC values compared to ChCl-based formulations. The most effective formulation, TA-TEAC, demonstrates consistent MIC values of 4.57 mg·mL^−1^ and MBC values of (9.14–18.23) mg·mL^−1^ against all tested bacterial strains, including resistant strains such as MRSA and *P. aeruginosa*. Particularly, GA-TEAC and LA-TEAC also show improved antibacterial activity compared to their ChCl-based formulations, with MIC values of (8.51–8.67) mg·mL^−1^ and MBC values of (17.35–34.69) mg·mL^−1^ (Table 4). Although both ChCl and TEAC are QACs, their toxic profiles differ significantly. ChCl is widely recognized for its low toxicity and biocompatibility and is frequently used in food, pharmaceutical, and biomedical formulations [65,66]. In contrast, TEAC exhibits moderate cytotoxicity, which is generally attributed to its steric bulk resulting from the four ethyl substituents surrounding the quaternary nitrogen center [37]. These structural features render the TEAC nitrogen cation more lipophilic and less capable of forming stabilizing hydrogen bonds compared to the choline cation. This increased lipophilicity promotes interaction with lipid bilayers, resulting into membrane insertion, disruption of membrane integrity, leakage of intracellular contents, and ultimately cell death [67]. This chemical behavior is analogous to that of surfactant compounds, many of which exhibit broad-spectrum cytotoxic effects due to non-specific membrane interactions [68]. Although the MIC values of the DES formulations are higher than those of conventional antibiotics (Table 4), this difference reflects the distinct non-antibiotic nature of DESs, which are primarily intended for local antimicrobial and antibiofilm applications. Antibiotics are low molecular weight compounds designed to selectively target specific intracellular bacterial processes, whereas DESs represent multicomponent, supramolecular systems whose antibacterial activity arises from combined physicochemical effects, including acidity, hydrogen bonding, membrane interaction, and disruption of the biofilm matrix. Consequently, DESs should not be expected to compete with antibiotics in terms of efficacy at the single-molecule level, but rather serve as non-antibiotic antimicrobial formulations suitable for local applications requiring high local concentrations, sustained contact, and antibiofilm activity. In this context, the observed MIC values of DESs remain consistent with their proposed use in topical formulations, coatings, and biofilm eradicating materials. The increased antibacterial efficacy of TEAC-based DESs formulations may be attributed to stronger interactions with bacterial membranes or improved solubilization of membrane lipids, despite these formulations having weaker HB frameworks, as evidenced by physicochemical data. In particular, the high density of hydroxyl and carboxyl groups in TA, combined with the rather hydrophobic and potentially membrane-disruptive nature of TEAC, likely contributes to the observed antibacterial efficacy. Moreover, the weaker hydrogen bonding framework formed by TEAC, compared to ChCl, suggests reduced stabilization of the acidic moieties of these αHAs. This reduced interaction likely increases the availability of free acid compounds, thereby enhancing the antibacterial effect through a combination of the intrinsic toxicity and antibacterial properties [69] of TEAC and the acidity of αHA. This highlights the importance of both the HBD and HBA structures for modulating the biological activity of a DES formulation.

To further evaluate the antibacterial efficacy of the DES formulations, time-kill kinetics against all four bacterial strains were assessed (SI Figure S4). All DESs exhibit time-dependent killing at MIC concentrations, with the rate varying between formulations. The time required to achieve a 3-log reduction in viable cells is summarized in Table 5. Overall, the killing kinetics followed the same structure-activity trends observed earlier for MIC and MBC values and hydrogen bonding capacity. GA-ChCl, GA-TEAC, LA-ChCl, LA-TEAC, and TA-ChCl show rapid bactericidal activity, as is typical for acidic DESs [18], achieving a 99.9% reduction within (2–6) h. This faster kinetics correlates with stronger or intermediate hydrogen-bonding frameworks and moderate acidity. In contrast, TA-TEAC consistently exhibits the slowest bactericidal action, requiring more than 6 h for all strains. Its reduced eradication rate, despite having the lowest pH of all DES formulations, indicates that acidity alone is not sufficient to determine the antibacterial efficacy of DES, and that the ability to form hydrogen bonds within the DES plays an essential mechanistic role.

**Figure 4:**
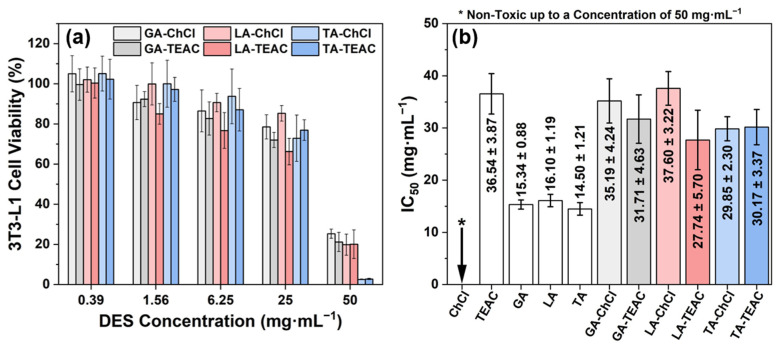
Cytotoxicity of individual components and DES formulations toward 3T3-L1 fibroblasts: (**a**) cell viability after 24-h exposure to increasing concentrations of DESs and (**b**) IC_50_ values of individual components and DES formulations. Data are presented as mean ± SD (n = 3).

The cytotoxicity results show a clear advantage of DES formation compared to individual αHAs (Figure 4). All DESs show a concentration-dependent reduction in the viability of 3T3-L1 fibroblasts and maintain cell viability above 70% at concentrations up to 25 mg·mL^−1^, indicating good cytocompatibility at moderate concentrations after 24 h exposure (Figure 4a). At a higher concentration of 50 mg·mL^−1^, cell viability dropped considerably. This effect is especially pronounced for DES formulations containing TA, which reduced the fibroblast viability below 5%. The IC_50_ values confirm these trends. TEAC and the pure αHAs are intrinsically cytotoxic with IC_50_ values between (14.50 ± 1.21) mg·mL^−1^ and (36.54 ± 3.87) mg·mL^−1^, whereas ChCl shows no toxicity at concentrations up to 50 mg·mL^−1^ (Figure 4b). Importantly, the DES formulations consistently displayed significantly higher IC_50_ values than individual αHAs, indicating that DES formation reduces cytotoxicity. This behavior can be explained by the formation of hydrogen bond networks in DESs. These partially stabilize the acidic components and reduce proton release compared to unbound acid molecules. These findings are well consistent with reports that acidic DESs exhibit increased cytotoxicity compared to non-acidic natural deep eutectic solvents (NADES) and that toxicity depends on the composition, concentration and structure of both HBD and HBA [26,66,71]. Moreover, the measured pH values correlate with biological responses: DES formulations with lower pH values generally exhibit stronger antibacterial activity and higher cytotoxicity, while less acidic DES formations display better fibroblast viability at a concentration of 50 mg·mL^−1^ (Figure 4a and Table 2). Taken together, these observations suggest that acidity is an important factor in the antibacterial efficacy and biocompatibility of these formulations. In addition, the number and spatial arrangement of hydroxyl and carboxyl groups in the α-hydroxy acids, as well as the hydrogen bonding capacity and lipophilicity of the HBA, further affect the overall biological response.

**Figure 5:**
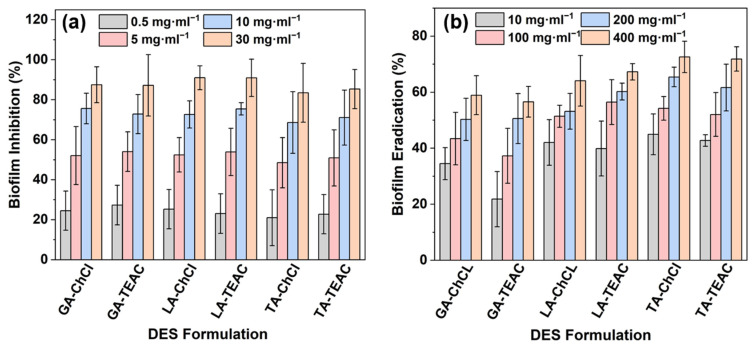
Anti-biofilm activity of the DES formulations investigated against *P. aeruginosa* at varying concentrations: (**a**) inhibition of biofilm formation and (**b**) eradication of pre-formed biofilms. Data are presented as mean ± SD (n = 3).

The anti-biofilm activity of the DES formulations was evaluated using two complementary assays for inhibiting biofilm formation and eradication of pre-formed biofilms. Due to its high clinical relevance, strong biofilm-forming ability, and intrinsic resistance to many conventional antibiotics, *P. aeruginosa* was used as the model strain [72,73]. Such a dual approach is consistent with the literature, which shows that DESs can both prevent biofilm formation and reduce or eradicate established biofilms [73,74]. All DES formulations display concentration-dependent inhibition of biofilm formation by *P. aeruginosa* (Figure 5a). At low concentrations of (0.5–5.0) mg·mL^−1^, partial inhibition is observed in all DES formulations, with efficacy increased markedly at 10 mg·mL^−1^ and 30 mg·mL^−1^. At the highest concentration tested, biofilm formation is almost completely suppressed, with several DES formations reaching inhibition of more than 90% (Figure 5a). Overall, the ability of DESs to prevent biofilm formation is consistent across formulations and correlates well with increasing concentration, indicating a robust inhibitory effect on early-stage biofilm formation [75].

In contrast, the eradication of mature biofilms requires significantly higher DES concentrations than those that are effective for prevention (Figure 5b). In the experiments, all DES formulations show moderate removal activity at (10–100) mg·mL^−1^ concentrations, with considerably stronger effects observed at 200 mg·mL^−1^ and 400 mg·mL^−1^. At the highest concentrations tested, biofilm is reduced by approximately (70–80)%, depending on the DES formulation. TA-based DESs with ChCl or TEAC achieve the highest eradication rates, closely followed by LA-based DES formulations. GA-based DESs are less effective at low and intermediate concentrations (10 mg·mL^−1^ and 100 mg·mL^−1^; Figure 5b). These results suggest that although all DESs possess biofilm-eradication properties, their capability to disrupt mature biofilms is more on the chemical formulation and requires higher concentrations compared to inhibiting biofilm formation.

In context with the MIC, MBC and IC_50_ data, these findings reveal several trends. The concentrations required to prevent biofilm formation are usually below the IC_50_ values for fibroblasts, which generally range from 20 mg·mL^−1^ to 40 mg·mL^−1^. This indicates a potentially favorable therapeutic window for applications aimed at preventing biofilm formation. Furthermore, the eradication of mature biofilms generally requires much higher concentrations that exceed the IC_50_ values for fibroblast. This limits the potential for systemic or prolonged exposure, but supports local and limited use where high DES concentrations can be tolerated. The strong activity observed with LA- and TA-based DES formulations may be partly due to their higher acidity, especially in the case of TA, which can enhance bactericidal mechanisms such as membrane disruption. However, acidity is likely one of several important determinants, including structural features of the HBD, type of HBA, and HB framework strength. The stabilization of acid functional groups via HBs in DESs may reduce the bioavailability of free protonated species and thus help to balance antibacterial activity with cytocompatibility. Overall, these results demonstrate that αHA-based DES formulations can be effective both for prevention and partially eradicating *P. aeruginosa* biofilms.

**Figure 6:**
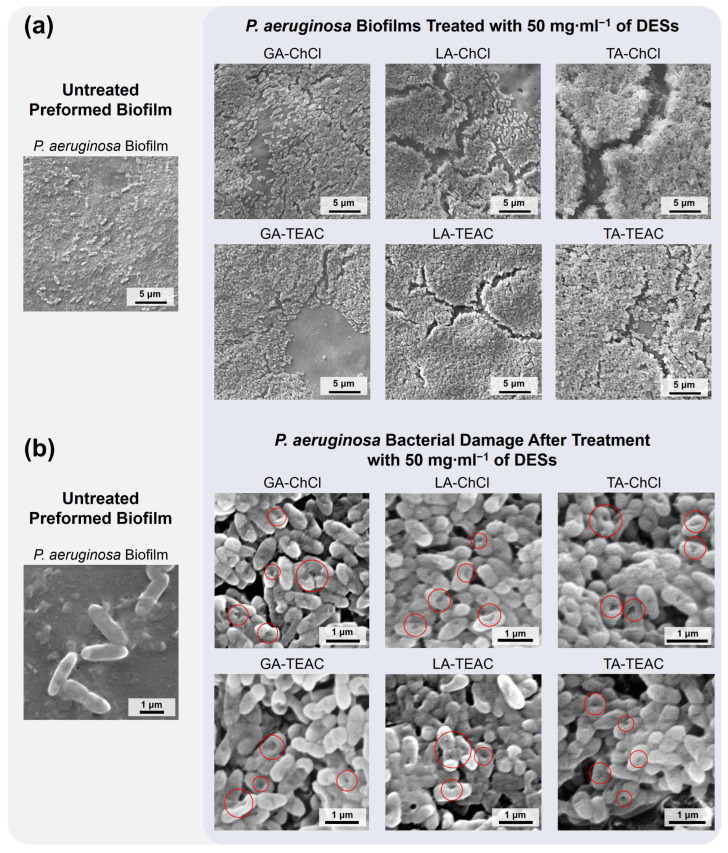
SEM micrographs of *P. aeruginosa* biofilms before and after treatment with the DES formulations investigated in this study at 50 mg·mL^−1^ reveals (**a**) disruption of biofilm structure and (**b**) damage to the bacterial cells (highlighted with red circles).

To explore the mechanism of action of DESs against *P. aeruginosa* biofilms, biofilm morphology and bacterial integrity were examined after treatment using scanning electron microscopy (SEM). Untreated biofilms show a dense and cohesive layer of tightly packed bacterial cells embedded in the extracellular matrix, which is characteristic of mature *P. aeruginosa* biofilms (Figure 6a) [76,77]. After treatment with 50 mg·mL^−1^ of DESs, all formulations display morphological disruption of the biofilm structure. Treated biofilms show visible thinning, fragmentation, and loss of biofilm continuity (Figure 6a). SEM and light microscopic studies in the literature provide visual evidence that disruption of the extracellular matrix leads to detachment and collapse of the biofilm architecture. For example, Powell et al. [78] studied biofilms of *P. aeruginosa* treated with alginate oligomers. SEM micrographs reveal a dose-dependent disruption of the extracellular polymeric substance (EPS): the cells are less embedded, matrix continuity is lost, and surface cracks and voids appear. In another study, Lam et al. [79] demonstrated that a branched polyethylenimine can bind to anionic components of the EPS biofilm of *P. aeruginosa* and induce matrix dissolution. Treated biofilms show structural collapse and fragmentation. These observations support the interpretation that morphological features such as cracks or detachments visible in SEM micrographs after treatment with DESs are consistent with EPS disruption [80]. DES formulations based on αHAs may denature EPS-associated proteins and depolymerize eDNA [81]. Additionally, the amphiphilic character of certain formulations (TEAC-based) can enhance the solubilization of EPS components. These combined effects are likely to compromise the cohesive properties of the extracellular matrix, leading to the morphological phenomena observed in the SEM micrographs.

SEM micrographs with higher-magnification of biofilms exposed to 50 mg·mL^−1^ of DESs reveal bacterial cells deformations, surface indentations, and localized ruptures (Figure 6b, indicated by red circles). Such damage is consistent with a direct interaction of the DES with the bacterial envelope, possibly mediated by acid-induced destabilization [23]. Similar morphological features have been reported previously for bacteria exposed to DESs of varying compositions [26,73]. These structures are commonly interpreted as evidence of damage to the cell envelope or a surface disintegration caused by a disruption in the integrity of the outer layer. These observations support the hypothesis that the antibacterial action of αHA-based DES formulations involves both the disruption of the biofilm matrix and direct damage to the bacterial envelope. It is important to note that the concentration used for SEM imaging corresponds to the lower end of concentrations that are effective in quantitative biofilm eradication assays and approaches the IC_50_ value. This suggests that structural damage to the biofilm occurs near to the upper tolerance limit of cytocompatibility. This confirms the assumption that these DESs may be best suited for surface applications where high local concentration can be achieved.

**Figure 7:**
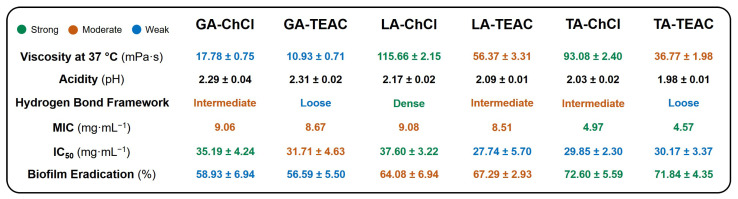
Summary of the research results in this study on the properties of deep eutectic solvents prepared of α-hydroxy acids and quaternary ammonium compounds.

A comparative overview of the physicochemical and biological properties of the six DES formulations examined is summarized in Figure 7. The overview reveals clear correlations between acidity, hydrogen bonding strength, and biological performance. ChCl-based formulations generally have higher viscosities and denser hydrogen bond frameworks, which is consistent with stronger intermolecular interactions. In contrast, TEAC-based DESs are less associated, which is reflected in their lower viscosities and “loose” hydrogen bond framework. The acidity of the DES formulations follows the order GA > LA > TA, with TA-containing DESs showing the lowest pH values, which correlates strongly with their enhanced antibacterial and biofilm-eradicating activity. Importantly, all DESs demonstrate IC_50_ values above 25 mg·mL^−1^, confirming acceptable cytocompatibility within the effective antibacterial range. Overall, the results show that the antibacterial efficacy of αHA-based DESs is primarily determined by acidity and hydrogen bonding capacity. In contrast, cytotoxicity remains controlled by the strength of hydrogen bonds and the chemical structure of the HBA.

## 4. Conclusions

The development of antibacterial deep eutectic solvents has been limited by a lack of understanding of how their molecular composition determine biological activity. In particular, the role of hydrogen bonds, acidity, and component structure in determining antibacterial efficacy and cytocompatibility has not yet been sufficiently studied. This study presents a systematic investigation of deep eutectic solvent formulations based on biocompatible α-hydroxy acids (glycolic acid, L-(+)-lactic acid and L-(+)-tartaric acid) and quaternary ammonium compounds (choline chloride and tetraethylammonium chloride) as potential antibacterial and antibiofilm agents. The results demonstrate that deep eutectic solvent formation significantly alters the biological properties of the individual components through supramolecular organization driven by hydrogen bonding. All deep eutectic solvents exhibited low pH values and broad-spectrum antibacterial activity against both Gram-positive and Gram-negative strains. Despite exhibiting weaker hydrogen bond frameworks, deep eutectic solvents based tetraethylammonium chloride showed enhanced antibacterial efficacy, which is likely due to the higher intrinsic toxicity of tetraethylammonium chloride and its limited ability to form stabilizing hydrogen bonds with α-hydroxy acids. A comparison with conventional antibiotics indicates that DES formulations require higher concentrations to inhibit bacterial growth. This difference is to be expected due to the fundamentally distinct, non-antibiotic nature of DESs and their supramolecular, physicochemical mechanism of action. Rather than competing with antibiotics in terms of efficacy, α-hydroxy acids–based DESs offer complementary advantages for localized antimicrobial and antibiofilm applications, where high local concentrations and sustained contact can be achieved.

The deep eutectic solvents effectively inhibited the formation of *P. aeruginosa* biofilms at sub-cytotoxic concentrations and demonstrated moderate eradication of preformed biofilms at higher concentrations. Morphological analysis confirmed that exposure to deep eutectic solvents led to the disruption of both the extracellular polymeric substance and the bacterial membranes, suggesting a dual mechanism of action. Importantly, the deep eutectic solvent formations reduced the cytotoxicity of the individual components, as evidenced by higher IC_50_ values compared to pristine α-hydroxy acids. This effect was particularly pronounced in deep eutectic solvent formulations based on choline chloride, probably due to the stabilization of acidic species by hydrogen bonds and the reduced availability of free acid molecules.

Together, these findings establish that α-hydroxy acid based deep eutectic solvents as tunable, biocompatible antimicrobial agents. The ability to combine strong antibacterial and antibiofilm activity with reduced cytotoxicity highlights their potential for biomedical applications such as wound dressings, surface coatings or skin antiseptics. Future studies should aim to translate these findings toward practical biomedical applications by evaluating the performance of these DES formulations in relevant in vivo models. Further optimization of DES composition could enable fine-tuning of acidity, viscosity, and hydrogen bond strength to balance antibacterial activity with cytocompatibility. Moreover, incorporating these DESs into polymeric matrices or gels may facilitate localized and sustained delivery to sites of infection, for example in wound dressings, implant coatings, or scaffolds for tissue engineering.

## Figures and Tables

**Table 1:** Compositions of the deep eutectic solvent (DES) formulations designed and investigated in this study. The table indicated the hydrogen bond donor (HBD) and the hydrogen bond acceptor (HBA) compounds, as well as their amount of substance (nHBD and nHBA) for the DES formulations prepared.

DES	HBD	HBA	nHBD (mol)	nHBA (mol)	Water (wt.%)
GA-ChCl	Glycolic Acid	Choline Chloride	2	1	18.3
GA-TEAC	Glycolic Acid	Tetraethylammonium Chloride			17.0
LA-ChCl	L-(+)-Lactic Acid	Choline Chloride			9.2
LA-TEAC	L-(+)-Lactic Acid	Tetraethylammonium Chloride			8.6
TA-ChCl	L-(+)-Tartaric Acid	Choline Chloride			14.6
TA-TEAC	L-(+)-Tartaric Acid	Tetraethylammonium Chloride			13.9

**Table 2:** Physicochemical properties of DES formulations based on αHAs and QACs. Physical-chemical properties of DES formulations based on αHAs and QACs. Presented by the values of dynamic viscosity, density, surface tension, pH value, and cold crystallization temperature (T_cc_).

DES Formation	Dynamic Viscosity * (mPa·s)	Density * (g·cm^−3^)	Surface Tension * (mN·m^−1^)	pH ^†^	T_cc_ (°C)
GA-ChCl	17.78 ± 0.75	1.16 ± 0.01	56.43 ± 0.11	2.29 ± 0.04	-
GA-TEAC	10.93 ± 0.71	1.10 ± 0.02	59.05 ± 0.08	2.31 ± 0.02	-
LA-ChCl	115.66 ± 2.15	1.16 ± 0.02	43.33 ± 0.01	2.17 ± 0.02	-
LA-TEAC	56.37 ± 3.31	1.09 ± 0.02	45.81 ± 0.04	2.09 ± 0.01	-
TA-ChCl	93.08 ± 2.40	1.26 ± 0.01	45.62 ± 0.23	2.03 ± 0.02	-
TA-TEAC	36.77 ± 1.98	1.17 ± 0.03	54.22 ± 0.10	1.98 ± 0.01	6.32 ± 1.22

* Dynamic viscosity, density and surface tension were measured at 37 °C; ^†^ pH values were determined in 100 mM aqueous solutions.

**Table 3:** Zones of inhibition caused by DES formulations against Gram-positive (*S. aureus* and MRSA) and Gram-negative (*E. coli* and *P. aeruginosa*) bacterial strains using an agar diffusion assay (n = 3).

DES	Zone of Inhibition (mm)			
	*S. aureus*	MRSA	*E. coli*	*P. aeruginosa*
Antibiotic *	32–36	15–17	13–14	14–16
GA-ChCl	19–21	19–20	18–20	17–18
GA-TEAC	16–19	19–20	17–18	17–18
LA-ChCl	21–22	18–19	19–20	20–21
LA-TEAC	15–16	17–18	14–16	15–16
TA-ChCl	16–19	17–18	16–18	16–18
TA-TEAC	20–21	19–20	19–20	20–21

* Ampicillin (25 mg) for *S. aureus*, MRSA, and *E. coli*; and ceftazidime (25 mg) for *P. aeruginosa*.

**Table 4:** Minimum inhibitory concentration (MIC) and minimum bactericidal concentration (MBC) values for the DES formulations tested towards Gram-positive bacteria strains (*S. aureus* and MRSA) as well as Gram-negative bacteria strains (*E. coli* and *P. aeruginosa*). The values correspond to the results obtained from three independent experiments (n = 3). Inter-assay variability did not exceed one dilution step.

		Antibacterial Properties			
DES Formulation	MIC MBC (mg·mL^−1^)	Bacterial Strain			
		Gram-Positive		Gram-Negative	
		*S. aureus*	*MRSA*	*E. coli*	*P. aeruginosa*
Antibiotic ^†^ [70]	MIC	<0.01	<0.01	<0.01	<0.01
GA	MIC MBC	4.97 19.85	4.97 19.85	4.97 19.85	4.97 19.85
LA	MIC MBC	2.80 44.20	2.80 88.30	5.50 44.20	2.80 44.20
TA	MIC MBC	3.91 15.63	3.91 15.63	3.91 15.63	3.91 15.63
ChCl	MIC MBC	- * -	- -	- -	- -
TEAC	MIC MBC	- -	- -	- -	- -
GA-ChCl	MIC MBC	9.06 36.25	9.06 36.25	9.06 18.13	9.06 18.13
GA-TEAC	MIC MBC	8.67 17.35	8.67 17.35	8.67 34.69	8.67 34.69
LA-ChCl	MIC MBC	9.08 34.40	9.08 34.40	9.08 34.40	9.08 34.4
LA-TEAC	MIC MBC	8.51 29.00	8.51 29.00	8.51 29.00	8.51 29.00
TA-ChCl	MIC MBC	4.97 9.92	4.97 9.92	4.97 9.92	4.97 9.92
TA-TEAC	MIC MBC	4.57 9.14	4.57 9.14	4.57 18.23	4.57 18.23

^†^ Ampicillin for *S. aureus*, MRSA and *E. coli*; and ceftazidime for *P. aeruginosa* according to EUCAST clinical breakpoint data. *—no bacterial inhibition.

**Table 5:** Time required to achieve a 99.9% reduction of Gram-positive (*S. aureus* and MRSA) and Gram-negative (*E. coli* and *P. aeruginosa*) bacteria strains at DES concentrations equal to 1 × MIC. Identical values were obtained in all independent experiments (n = 3).

DES Formulation	Time to Eradicate 99.9% of Bacteria (h)			
	Bacterial Strain			
	Gram-Positive		Gram-Negative	
	*S. aureus*	MRSA	*E. coli*	*P. aeruginosa*
GA-ChCl	6	6	6	4
GA-TEAC	4	4	6	4
LA-ChCl	6	4	6	6
LA-TEAC	6	2	6	4
TA-ChCl	6	2	6	6
TA-TEAC	24	24	24	24

## Data Availability

The data that support the findings of this study are available from the corresponding authors upon reasonable request.

## Supplementary Materials

The following supporting information can be downloaded at: https://www.mdpi.com/article/10.3390/pharmaceutics18010016/s1, Figure S1: Substrates fabricated for biofilm growth: (a) single 3D-printed substrate prepared via photolithographic 3D-printing, (b) substrates attached to the lid of a 24-well plate and (c) substrates immersed in nutrient medium during incubation. Figure S2: Visual appearance of the deep eutectic solvents (DESs) prepared after 24 h of storage at room temperature. From left to right: glycolic acid-choline chloride (GA-ChCl), lactic acid-choline chloride (LA-ChCl), tartaric acid-choline chloride (TA-ChCl), glycolic acid-tetraethylammonium chloride (GA-TEAC), lactic acid-tetraethylammonium chloride (LA-TEAC), and tartaric acid-tetraethylammonium chloride (TA-TEAC). Figure S3: Inhibition zones of bacterial growth via the agar diffusion method of all DES formations against Gram-positive bacterial strains (*S. aureus* and MRSA) and Gram-negative bacterial strains (*E. coli* and *P. aeruginosa*). Figure S4: Time–kill curves of DES formulations against (a) *Staphylococcus aureus*, (b) methicillin-resistant *Staphylococcus aureus* (MRSA), (c) *Escherichia coli*, and (d) *Pseudomonas aeruginosa* at 1 × MIC concentrations. The data are presented as mean ± SD (n = 3).
