# Supplementary material for: Tailorable Antibacterial Activity and Biofilm Eradication Properties of Biocompatible α-Hydroxy Acid-Based Deep Eutectic Solvents
Source: Pharmaceutics. 2025 Dec 22;18(1):16. doi: 10.3390/pharmaceutics18010016 (PMC12845313; doi:10.3390/pharmaceutics18010016)
Supplement: Supplementary file 1 [file pharmaceutics-18-00016-s001.zip › pharmaceutics-3972400-supplementary.pdf]

pharmaceutics

MDPI

Article

Supporting Information of: Tailorable antibacterial activity and biofilm eradication properties of biocompatible α-hydroxy acid-based deep eutectic solvents

Gleb Dubinenko[1,*], Elena Senkina[1], Ksenia Golovina[1], Alexandra Myshova[1], Olga Igumnova[1], Evgenii Plotnikov[2,3,4], Arsalan Badaraev[1], Sven Rutkowski[1,*], Victor Filimonov[5], Sergei Tverdokhlebov[1,*]

Supporting Figures

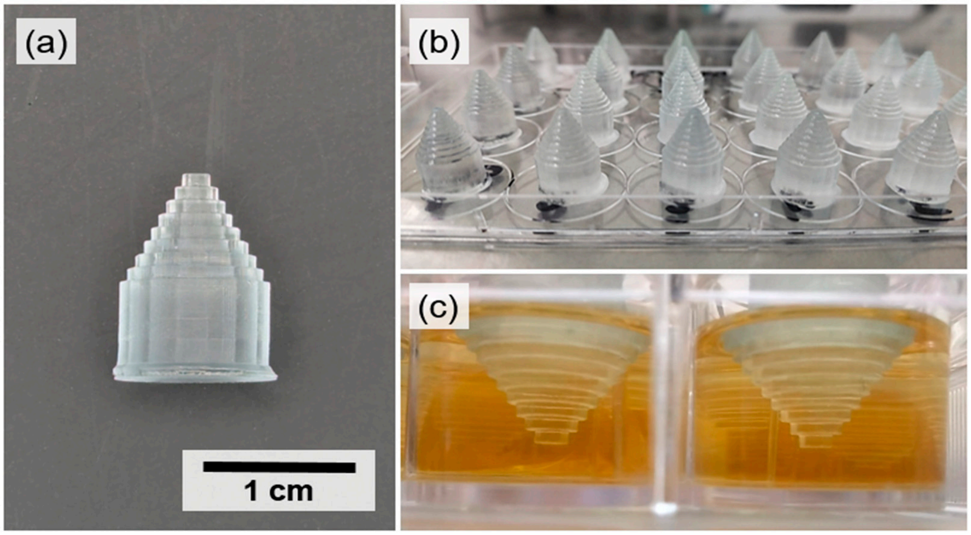

Figure S1. Substrates fabricated for biofilm growth: (a) single 3D-printed substrate prepared via photolithographic 3D-printing, (b) substrates attached to the lid of a 24-well plate and (c) substrates immersed in nutrient medium during incubation.

 https://doi.org/10.3390/pharmaceutics18010016

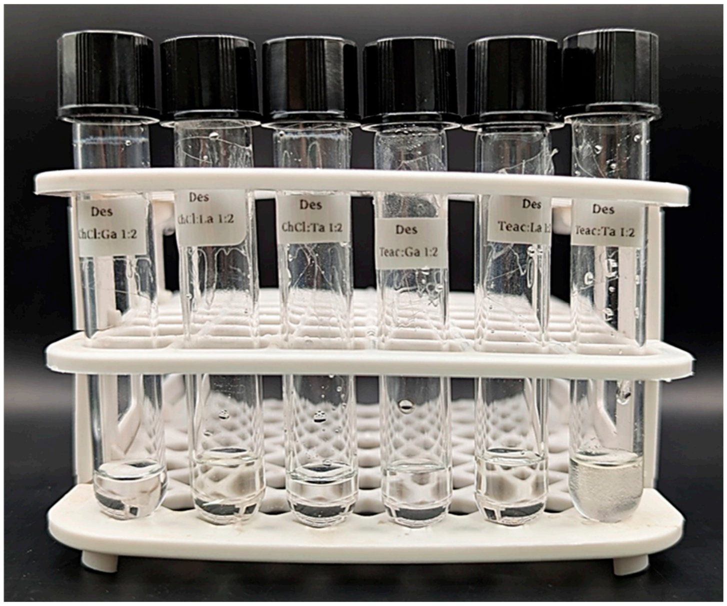

Figure S2. Visual appearance of the deep eutectic solvents (DESs) prepared after 24 hours of storage at room temperature. From left to right: glycolic acid-choline chloride (GA-ChCl), lactic acid-choline chloride (LA-ChCl), tartaric acid-choline chloride (TA-ChCl), glycolic acid-tetraethylammonium chloride (GA-TEAC), lactic acid-tetraethylammonium chloride (LA-TEAC), and tartaric acid-tetraethylammonium chloride (TA-TEAC).

https://doi.org/10.3390/pharmaceutics18010016

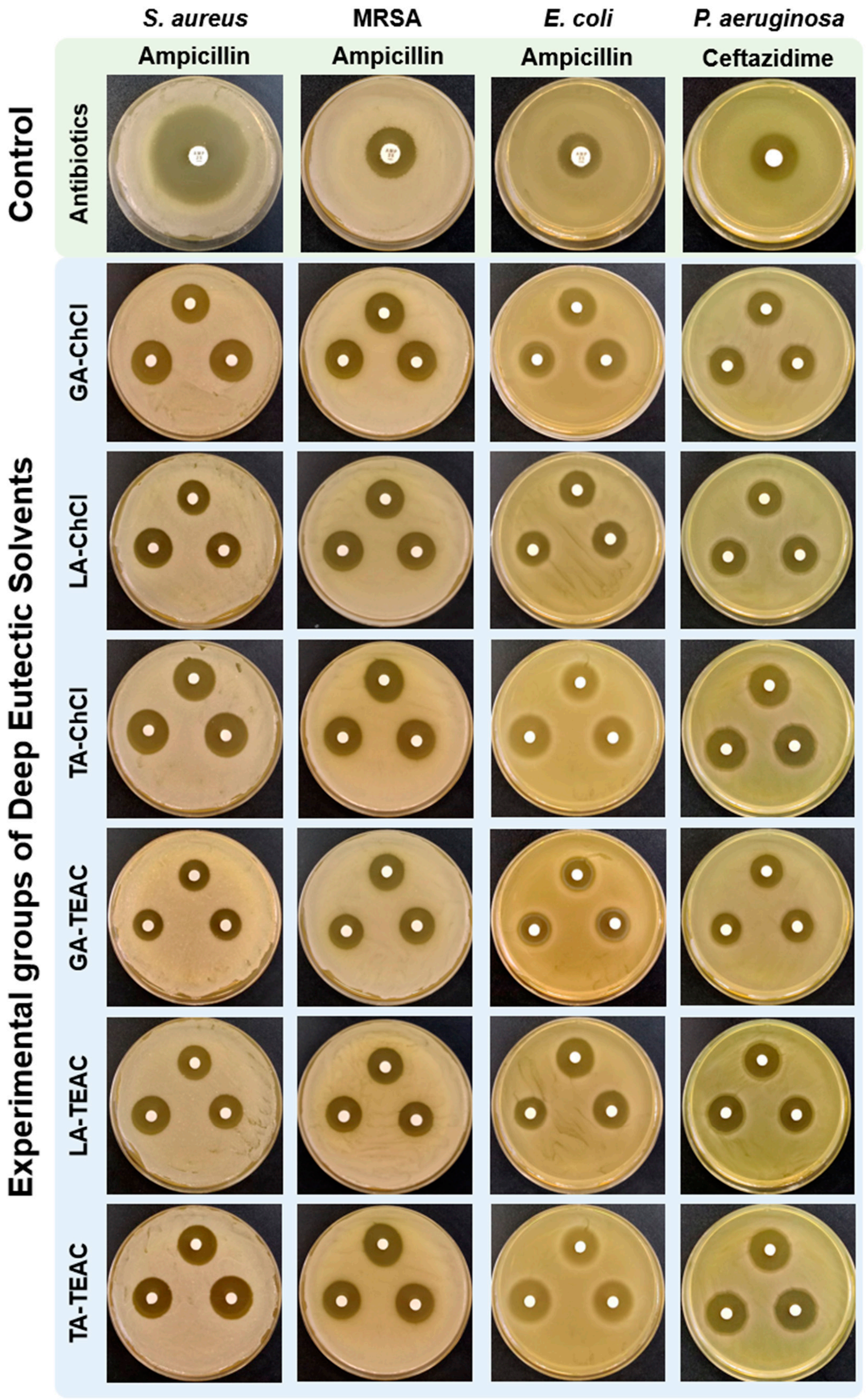

Figure S3. Inhibition zones of bacterial growth via the agar diffusion method of all DES formations against Gram-positive bacterial strains (*S. aureus* and MRSA) and Gram-negative bacterial strains (*E. Coli* and *P. aeruginosa*).

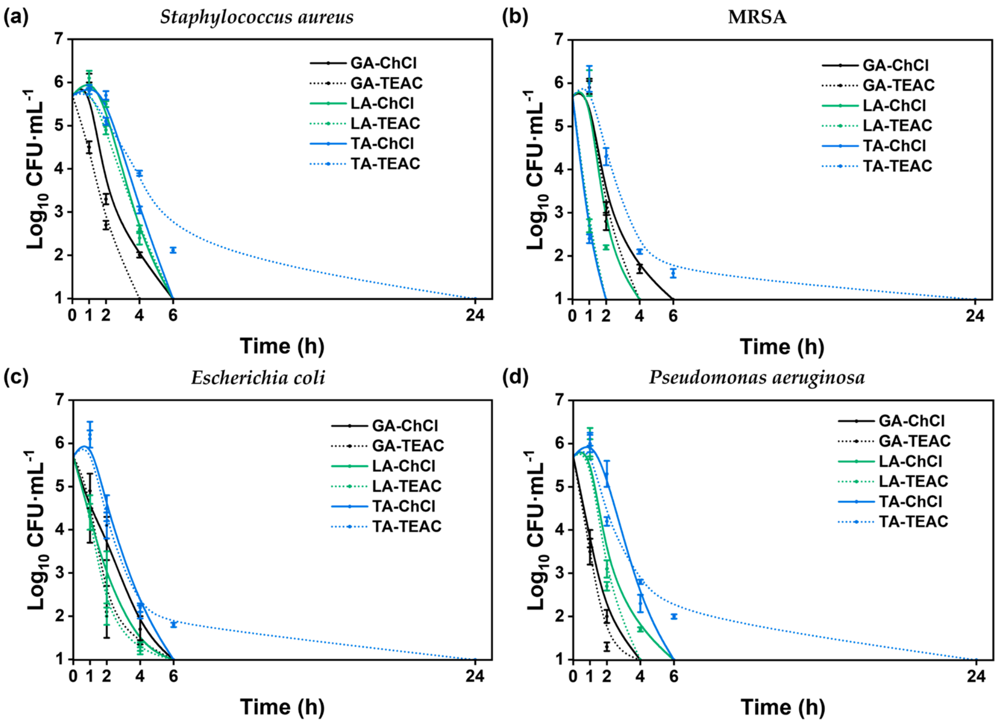

Figure S4. Time–kill curves of DES formulations against (a) *Staphylococcus aureus*, (b) methicillin-resistant *Staphylococcus aureus* (MRSA), (c) *Escherichia coli*, and (d) *Pseudomonas aeruginosa* at 1 × MIC concentrations. The data are presented as mean ± SD (n = 3).

https://doi.org/10.3390/pharmaceutics18010016
